# Antenatal corticosteroids reduce neonatal mortality in settings without assisted ventilatory support: a retrospective cohort study of early preterm births on the Thailand-Myanmar border

**DOI:** 10.12688/wellcomeopenres.19396.1

**Published:** 2023-05-18

**Authors:** Humayra Aisha Bashir, Daphne Lufting-Leeffrers, Aung Myat Min, Htun Htun Win, Nay Win Tun, Tha Gay Wah, Mary Ellen Gilder, Moo Kho Paw, Verena I. Carrara, Aronrag Meeyai, Adeniyi Kolade Aderoba, François Nosten, Mechthild M. Gross, Rose McGready

**Affiliations:** 1 Shoklo Malaria Research Unit, Mahidol-Oxford Tropical Medicine Research, Faculty of Tropical Medicine, Mahidol University, Salaya, Nakhon Pathom, Thailand; 2 Centre for Tropical Medicine & Global Health, Nuffield Department of Medicine, University of Oxford, Oxford, England, UK; 3 Midwifery Research and Education Unit, Hannover Medical School, Hannover, Germany; 4 Department of Family Medicine, Chiang Mai University, Chiang Mai, Chiang Mai, Thailand; 5 Institute of Global Health, Faculty of Medicine, Universite de Geneve, Geneva, Geneva, Switzerland; 6 University of Medical Sciences Teaching Hospital, Akure, Ondo, Nigeria

**Keywords:** Preterm birth, Early preterm birth, Antenatal corticosteroids (ACS), Dexamethasone, Low resource settings, neonatal mortality, assisted ventilation, CPAP

## Abstract

**Background:** Prematurity is the highest risk for under-five mortality globally. The aim of the study was to assess the effect of antenatal dexamethasone on neonatal mortality in early preterm in a resource-constrained setting without assisted ventilation.

**Methods:** This retrospective (2008-2013) cohort study in clinics for refugees/migrants on the Thai-Myanmar border included infants born <34 weeks gestation at home, in, or on the way to the clinic. Dexamethasone, 24 mg (three 8 mg intramuscular doses, every 8 hours), was prescribed to women at risk of preterm birth (28 to <34 weeks). Appropriate newborn care was available: including oxygen but not assisted ventilation. Mortality and maternal fever were compared by number of doses (complete: three, incomplete: one or two, or no dose). A sub-cohort participated to neurodevelopmental testing at one year.

**Results:** Of 15,285 singleton births, 240 were included: 96 did not receive dexamethasone and 144 received one, two or three doses (56, 13 and 75, respectively). Of live born infants (n=233), early neonatal and neonatal mortality/ 1,000 livebirths (95%CI) with complete dosing was 141 (78–240) and 304 (191–448); compared to 292 (210–389) and 521 (407–633) with no dose. Compared to complete dosing, both incomplete and no dexamethasone were associated with elevated adjusted ORs 4.09 (1.39 to 12.00) and 3.13 (1.14 to 8.63), for early neonatal death. By contrast, for neonatal death, while there was clear evidence that no dosing was associated with higher mortality, adjusted OR 3.82 (1.42 to 10.27), the benefit of incomplete dosing was uncertain adjusted OR 1.75 (0.63 to 4.81). No adverse impact of dexamethasone on maternal fever or neurodevelopmental scores was observed.

**Conclusions:** Neonatal mortality reduction is possible with complete dexamethasone dosing in pregnancies at risk of preterm birth in settings without capacity to provide assisted ventilation.

## Lay summary

Prematurity is still the leading cause of under-five mortality and neonatal deaths globally. Antenatal corticosteroids (ACS), such as dexamethasone, have been used widely to decrease preterm mortality and morbidity. However, evidence of their impact without assisted ventilation in low-resource settings remains limited. We studied the effect of different doses of dexamethasone prescribed to women at risk of preterm birth between 28 to less than 34 weeks gestation on early neonatal and neonatal mortality in clinics at the Thai-Myanmar border. Results from our study suggest that complete dosing of dexamethasone could significantly reduce neonatal mortality in settings without basic assisted ventilation, such as continuous positive airway pressure (CPAP). We found no adverse effect of dexamethasone on maternal fever or neurodevelopmental scores.

## Introduction

Globally preterm birth (PTB), before 37 weeks gestational age, is the leading cause of under under-five mortality ^1^. The burden of mortality and morbidity before five years of age due to PTB is profound; affecting one million of 15 million newborns annually ^2^. Deaths are largely attributable to the lower end of survivable estimated gestational age from respiratory distress syndrome particularly in low-income settings ^3– 5^. Risk of lifelong adverse outcomes are also increased, for example, bronchopulmonary displasia, cerebral palsy, hearing impairments, intellectual or mortor disability and retinopathy of prematurity ^6^.

Antenatal corticosteroids (ACS) at less than 34 weeks of gestation are recommended to reduce preterm mortality and morbidity ^7– 9^ and not indicated from 34 to less than 37 weeks ^10^. ACS reduce neonatal respiratory distress syndrome by enhancing the production of surfactant binding proteins, foetal lung antioxidant enzymes and morphological development of type I and II pneumocytes, through which premature foetal lung function is improved ^11^. For low- and middle-income countries dexamethasone is common, inexpensive and easy to store.

The generalizability of studies conducted in high resource hospitals with neonatal intensive care facilities (surfactant, continuous positive airway pressure (CPAP) and assisted ventilation) to low resource settings, where 81.8% of the global burden of PTBs occur, has been tested *via* a series of trials ^12^. The cluster randomized Antenatal Corticosteroid Trial (ACT) ^13^ in six countries (Argentina, Guatemala, India, Kenya, Pakistan, and Zambia) contrary to the hypothesis, found an excess of 3.5 neonatal deaths for every 1,000 women exposed and maternal infections also increased. This resulted in the World Health Organization (WHO) modifying its guidelines for ACS for low resource settings ^14^. The Antenatal Corticosteroids for Improving Outcomes in preterm Newborns (ACTION) trial ^15^ followed and was intentionally placebo controlled to assess safety and efficacy of ACS in at risk women at 26 to <34 weeks of gestation in Bangladesh, India, Kenya, Nigeria, and Pakistan. Dexamethasone was associated with a significantly lower risk of neonatal death alone than the use of placebo, without an increase in the incidence of possible maternal bacterial infection. Furthermore, in a cost-effectiveness analysis of the trial, dexamethasone was cost saving when compared with no intervention ^16^.

In South-East Asia a retrospective descriptive study in four countries, Indonesia, Malaysia, the Philippines and Thailand observed variable ACS administration rates, 9–73%, highest in Thailand, with ACS associated with a reduction in stillbirth and neonatal mortality ^17^.

The aforementioned trials included outcomes in neonates with access to assisted ventilation: surfactant, CPAP and assisted ventilation ^13, 15, 17^. We aimed to determine the impact of ACS on early neonatal and neonatal mortality in infants born 28 to <34 weeks gestation on the Thailand-Myanmar border, in clinics with a neonatal intensive care unit but without capacity for assisted ventilation.

## Methods

### Ethics

Ethical approval for retrospective analysis of hospital records at the Shoklo Malaria Research Unit was under the guidance of the Oxford University Ethics Committee (OXTREC: 28-09, 6 ^th^ May 2009) and the local community advisory board in Mae-Sot, Thailand (TCAB-12/2/2015, 15 ^th^ August 2015). The ethical committee approved the protocol for the retrospective analysis of hospital records in the absence of consent due to the complexity of finding patients and the fact that all records were anonymised. Data collection from hospital records started in January 2021.

### Study design

A retrospective cohort study in a setting where ACS (dexamethasone) use is prescribed for refugee and migrant women with a risk of PTB at 28 to <34 weeks gestational age.

### Setting

The Shoklo Malaria Research Unit (SMRU) on the Thailand-Myanmar border is a field-based research organization, providing humanitarian care for refugees and migrants since 1986. Long standing neglect of the health system in Myanmar leaves swathes of the population without health services. Maela Camp is the largest of a number of camps based along the Thailand-Myanmar border providing shelter for refugees (estimated at 140,000) from Myanmar since 1984 in one of the most protracted refugee situations globally. An estimated 200,000 migrants from Myanmar work in Tak Province, Thailand, and live in sub-optimal and semi-permanent accommodation.

Rates of homebirth in Myanmar particularly in rural areas are high ^18, 19^ and SMRU has encouraged women to birth in unit facilities, resulting in a reversal from three quarters born at home in 1986, to more than three quarters being born in SMRU facilities by 2015 ^20^.

SMRU provides integrated care services including antenatal, birth, postnatal, and infant care, in the local languages (Sgaw Karen, Poe Karen, Burmese). Neonatal intensive care has been available in the refugee camp from 2008 ^21^ and in migrant camps from 2009 in Wang Pha and 2010 in Maw Ker Thai. Medics, midwives and nurses are assisted in the clinical work by expatriate doctors and quality of care has been assessed ^22^. Malaria control relies on antenatal early diagnosis and treatment to prevent mortality and reduce the adverse perinatal effects of infection ^23, 24^. HIV and syphilis are low in this setting with screening offered at the first consultation ^25^.

Approximately 2,000 women birth at SMRU clinics annually in clinics that can provide the seven signal functions of Basic Emergency Obstetric and Newborn Care: parenteral administration of an oxytocic, antibiotics and anticonvulsants, removal of retained products of conception, assisted vaginal birth including breech birth, immediate resuscitation of the newborn using a bag and mask for infants ≥28 weeks’ gestation, and screened blood transfusions. In these midwife led units staff are trained in emergencies with the Advanced Life Support in Obstetrics course ^26^. Women who require caesarean section for obstetric indications such as placenta praevia are referred to the nearest Thai Hospital.

### Ultrasound

Various scanners including Toshiba Powervision 7000, Dynamic Imaging (since 2001), Fukuda Denshi UF 4100, and General Electric Voluson-1 have been used locally to determine gestational age ^27– 29^. Women are routinely offered two scans: at booking to determine viability, number of foetus and gestation, regardless of how far progressed the pregnancy is, but preferably between eight and 14 weeks; and at 22 (18–24) weeks to reassess viability, measure foetal biometry and major abnormalities and determine placental location. Repeat scans for clinical indications are done as required. Late presenters to ANC get an ultrasound and the newborn also has a Dubowitz Assessment of gestational age, in use since 1993 with trained staff and annual quality control ^30^.

### Dexamethasone

The protocol for preterm labour recommends dexamethasone 24 mg (three doses of 8 mg intramuscular every eight hours) if gestation is 28 to <34 weeks, but in practice dexamethasone was occasionally given prior to 28 weeks. Repeat doses of dexamethasone were not given. All women with threatened preterm labour were screened for infection: malaria (Giemsa stained blood smear), urinary tract infection (urine stick and sediment), respiratory tract infection (lung auscultation and history of symptoms), with a vaginal swab for microscopy (wet prep for identification of trichomonas, candida). A sterile speculum examination is conducted to check for signs of infection, cervical dilation, and the presence of ruptured membranes by liquor pooling, or umbilical cord prolapse. Tocolytic (nifedipine) was prescribed when indicated. Magnesium sulphate for neuroprotection was not provided.

### Neonatal intensive care

The establishment and impact of a neonatal intensive care unit in this setting has been described previously ^21^. Appropriate care as prescribed in high-income settings for late preterm neonates, was available in the neonatal intensive care unit *i.e.*, regular check of vital signs, regular (breast)feeding, stable body temperature, monitored oxygen saturation and intravenous antibiotics if required. Respiratory support involved nasal or mask oxygen, apnoea mats, pulse oximetry, and intravenous aminophylline in place of oral caffeine (due to unavailability).

Staff were trained in newborn resuscitation and a study in this setting reported normal neurodevelopmental outcomes at one year of age following basic resuscitation at birth, compared to low-risk newborns who did not need resuscitation ^31^. Care is guided by local protocols available and used at each SMRU site. Extreme PTB (<28 weeks) infants are provided with palliative care and parents are involved and counselled in the process ^32^. Tertiary care referral is limited due to prohibitive costs.

### Neurodevelopment

The Shoklo Developmental Test at 12–14 months of age was used to assess neurodevelopment in a sub-cohort of infants enrolled in a previously approved birth cohort being conducted during the timeframe of this study ^33^. This test evaluates four different aspects of development with each item scored as pass/fail basis: coordination, speech, social interaction and milestone. Behaviour during the test (relation to the tester, interest towards the test, emotional status) was also evaluated.

### Eligibility for inclusion

Birth records from 2008 were captured in real time using an application developed by Technology Sans Frontières (TSF). Further verification of the major data (registration, partogram, inpatient admissions, birth outcome, neonatal intensive care unit charts) was possible from paper-based charts, including a PTB checklist. Records from 2008-2013 were reviewed if they were: i) preterm (28 to <34 weeks), and if ii) dexamethasone administration was confirmed from clinic files; in this way capturing all possible dexamethasone eligible cases of birth from 28 weeks.

Exclusion criteria included referral to the Thai hospital for birth; and when dexamethasone was not indicated, such as *e.g.*, detection of a severe congenital abnormality, or because there was no foetal heartbeat, or dexamethasone was given <26 weeks.

### Definitions

PTB included birth from 28 to <37 weeks of life, although in this series all were early preterms <34 weeks; stillbirth included infants of 28 weeks gestation with no signs of life; early neonatal mortality was death of a live born infant within the first seven days of life, neonatal mortality was death of a live born infant within the first 28 days of life. Complete dexamethasone refers to three doses, and incomplete to one or two doses. Congenital abnormality included newborns with an anomaly detected by ultrasound or on routine surface examination of the newborn including cardiac auscultation by trained staff and confirmed by a doctor.

### Statistical analysis

Categorical data were reported as proportion and compared using the Chi-squared test or Fishers Exact Test for cell values <5. Median and interquartile range [IQR] described continuous data, such as gestation, and compared using the Mann-Whitney U test, with univariate associations quantified using logistic regression. To evaluate the role of dexamethasone on early neonatal and neonatal mortality, all significant risk factors for zero and three doses were combined into one logistic regression model, including congenital abnormality, gestation at birth, maternal smoking and pre-eclampsia. Pre-eclampsia was included in the multivariable model in concordance with the literature as an important risk factor, and birthweight was not included due to collinearity with gestational age. Neurodevelopmental scores for complete or any dexamethasone were compared to zero dexamethasone and adjusted for gestational age at birth. Data were analysed using IBM SPSS Statistics (RRID:SCR_016479) version 27 (IBM SPPS, Armonk, NY, USA).

## Results

**Figure 1.**
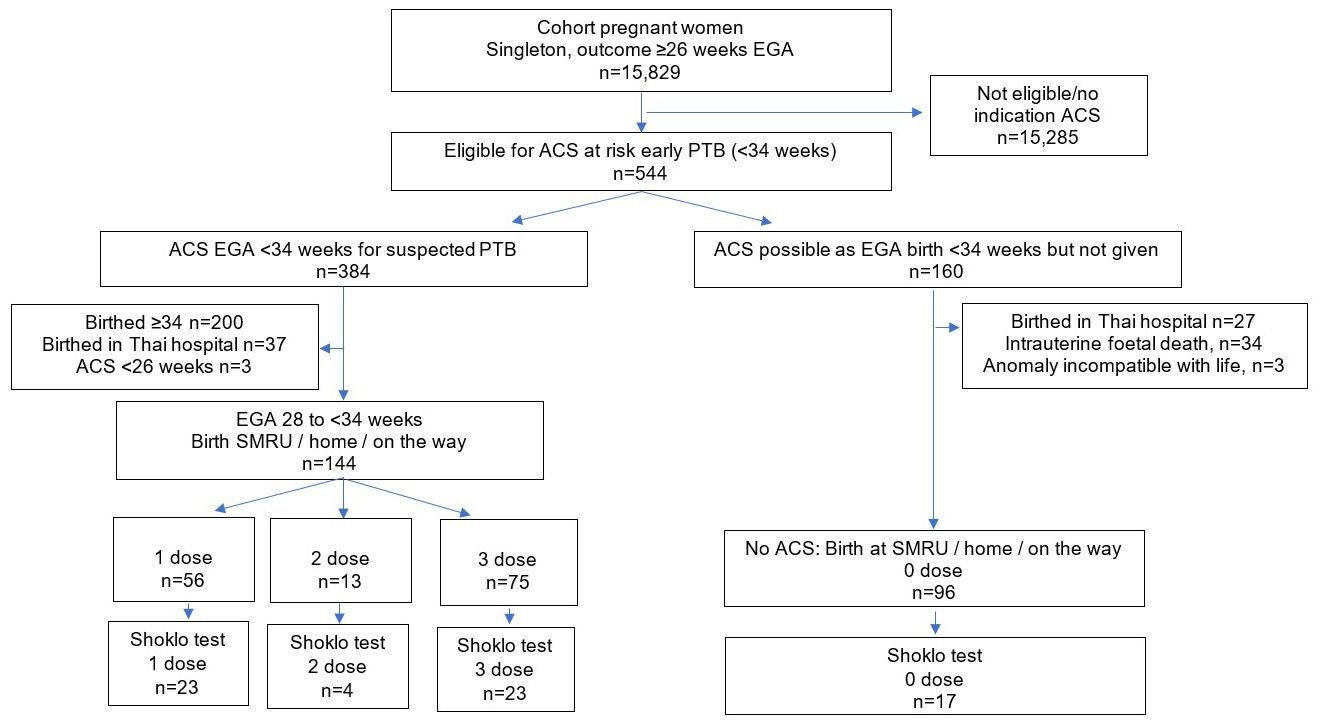
Study flow chart. EGA, estimated gestational age; ACS, antenatal corticosteroids; PTB, preterm birth; SMRU, Shoklo Malaria Research Unit.

Of 15,829 pregnant women with singleton births between January 2008 to December 2013 there were 3.4% (n=544) eligible for dexamethasone ( Figure 1). The final cohort included 240 women who birthed at the clinic, at home or on the way to the clinic with 60.0% (n=144) receiving at least one dose of dexamethasone, and 40.0% (n=96) where it was indicated (estimated gestational age <34 weeks) but not received ^5^.

### Baseline characteristics of the study population

**Figure 2.**
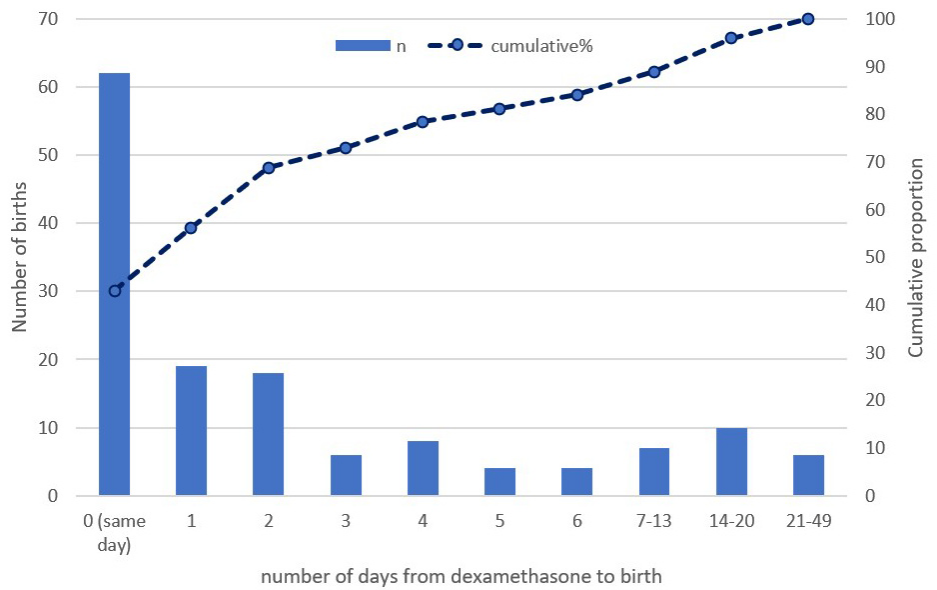
Dexamethasone to delivery interval (n=144).

The median maternal age was 22 years, almost half (43.8%, 105/240) were primigravidae, 47.5% (114/240) attended first ANC visit in first trimester and most women had ultrasound confirmed gestation (84.4%, 204/244) ( Table 1). Pre-eclampsia was significantly higher in the complete dexamethasone group ( Table 1). The majority, 84.0% (121/144) birthed within seven days of receiving dexamethasone ( Figure 2), with a longer interval from administration to birth in those who received a complete course ( Table 1).

**Table 1.** Maternal characteristics and dexamethasone doses.

Characteristics		Dexamethasone doses					P-value		
	Cohort	0 dose	1 dose	2 doses	1 or 2 doses	3 doses	3 doses *versus* 0 dose	1-2 doses *versus* 0 dose	3 doses *versus* 1-2 dose
	n=240	n=96	n=56	n=13	n=69	n=75			
Age, years	22 [19-28]	23 [19-30]	21 [18-25]	21 [19-28]	21 [18-26.0]	23 [19-30]	0.628	0.060	0.033
Primigravida	105 (43.8)	34 (35.4)	34 (60.7)	6 (46.2)	40 (58.0)	31 (41.3)	0.433	0.005	0.066
Refugee (not migrant)	131 (54.6)	45 (46.9)	34 (60.7)	10(76.9)	44 (63.8)	42 (56.0)	0.281	0.040	0.396
Smoker	46 (19.2)	30 (31.3)	4 (7.1)	1 (7.7)	5 (7.2)	11 (14.7)	0.012	<0.001	0.190
Literate ^^^	76/147 (51.7)	19/48 (39.6)	27/44 (61.4)	4/9 (44.4)	31/53 (58.5)	26/46 (56.5)	0.148	0.074	1.000
History Preterm birth	31 (12.9)	12 (12.5)	8 (14.3)	2 (15.4)	10 (14.5)	9 (12.0)	1.000	0.817	0.806
History NND	81 (33.8)	33 (34.4)	14 (25)	4 (30.8)	18 (26.1)	30 (40.0)	0.523	0.307	0.081
Anaemic 1 ^st^ HCT	33 (13.8)	18 (18.8)	5 (8.9)	1 (7.7)	6 (8.7)	9 (12.0)	0.292	0.078	0.592
**Pregnancy** ** morbidity**									
Malaria	33 (13.8)	19 (19.8)	6 (10.7)	0	6 (8.7)	8 (10.7)	0.139	0.077	0.782
Pre-eclampsia	11 (4.6)	1 (1.0)	2 (3.6)	0	2 (2.9)	8 (10.7)	0.006	0.378	0.064
APH	11 (4.6)	3 (3.1)	3 (5.4)	1 (7.7)	4 (5.8)	4 (5.3)	0.365	0.323	0.593
Birth ≤7 days ACS	n.a.	n.a.	56 (100.0)	13 (100.0)	69 (100.0)	52 (69.3)	n.a.	n.a.	<0.001

Data are presented as the Median, interquartile range [IQR], or n (%) unless otherwise stated; p-value- Chi-squared test or Fisher’s exact test if cell count<5, medians by Mann-Whitney U test Abbreviations: ACS antenatal corticosteroids, ANC antenatal care, APH antepartum haemorrhage, HCT haematocrit, n.a. not applicable, NND neonatal death. ^Literacy (self-reported) routinely collected from 2010.

### Birth outcomes

Overall, 78.3% (188/240) of the newborns were delivered at SMRU clinics and the majority were delivered following standard vaginal birth (88.8%, 213/240) ( Table 2). A small proportion of infants were stillborn (2.9%, 7/240), but foetal heartbeat was present before dexamethasone was prescribed ( Table 2).

**Table 2.** Neonatal characteristics and dexamethasone doses.

Variables		Dexamethasone doses					P-value		
	Cohort	0 dose	1 dose	2 dose	1 or 2 doses	3 doses	3 *versus* 0 dose	1-2 *versus* 0 dose	3 *versus* 1-2 dose
	n=240	n=96	n=56	n=13	n=69	n=75			
**Place of birth**							<0.001	<0.001	0.056
SMRU clinic	188 (78.3)	50 (52.1)	56 (100)	13 (100)	69 (100)	69 (92.0)			
Home	45 (18.8)	40 (41.7)	0	0	0	5 (6.7)			
On the way to clinic	7 (2.9)	6 (6.3)	0	0	0	1 (1.3)			
Male	146/237 (61.6)	55/93 (59.1)	39 (69.6)	10 (76.9)	49 (71.0)	42 (56.0)	0.754	0.137	0.083
Congenital Abnormality	13/237 (5.5)	6/93 (6.5)	3 (5.4)	n.a.	3 (4.3)	4 (5.3)	1.000	0.415	0.546
EGA birth, weeks, Median [IQR]	32.1 [30.4-33.2]	32.3 [30.3-33.2]	32.1 [30.5-33.3]	31.5 [29.7-32.3]	31.6 [30.4-33.0]	32.2 [30.2-33.2]	0.616		
EGA by US, n (%)	206 (85.8)	83 (86.5)	49 (87.5)	12 (92.3)	61 (88.4)	62 (82.7)	0.525	0.815	0.355
**PTB group**							0.823	0.364	0.269
PTB 28-29 weeks	43 (17.9)	16 (16.7)	9 (16.1)	3 (23.1)	12 (17.4)	15 (20.0)			
PTB 30-31 weeks	62 (25.8)	23 (24.0)	18 (32.1)	5 (38.5)	23 (33.3)	16 (21.3)			
PTB 32-33 weeks	135 (56.3)	57 (59.4)	29 (51.8)	5 (38.5)	34 (49.3)	44 (58.7)			
**Type of delivery**							0.548	0.327	0.409
Cephalic flexed vaginal birth	213 (88.8)	87 (90.6)	47 (83.9)	12 (92.3)	59 (85.5)	67 (89.3)			
Breech/Face	26 (10.8)	8 (8.3)	9 (16.1)	1 (7.7)	10 (14.5)	8 (9.7)			
Forceps	1 (0.4)	1 (0.4)	0	0	0	0			
Weighed in 72 hrs	225/231 (97.4)	83/88 (94.3)	56 (100)	13 (100)	69 (100)	73/74 (98.6)	0.151	0.053	0.517
Birth weight *, kg Median [IQR]	1.65 [1.36- 1.90] n=206	1.66 [1.30- 19.5] n=77	1.72 [1.47- 1.95] n=51	1.66 [1.22- .85] n=12	1.72 [1.42- 1.91] n=63	1.66 [1.30- 1.88] n=66	0.729	0.823	
Apgar < 7 at 5 min	14/182 (7.7)	6/51 (11.8)	2/53 (3.8%)	0	2/65 (3.1)	6/66 (9.1)	0.761	0.072	0.142
Stillbirth	7 (2.9)	0	2 (3.6)	1 (7.7)	3 (4.3)	4 (5.3)	0.035	0.071	0.546
Early Neonatal death	56/233 (24.0)	28/96 (29.2)	13/54 (24.1)	5/12 (41.7)	18/66 (27.3)	10/71 (14.1)	0.025	0.860	0.061
Neonatal death **	69/168 (41.1)	37/71 (52.1)	13/40 (32.5)	5/11 (41.5)	18/51 (35.3)	14/46 (30.4)	0.023	0.096	0.669

Data are presented as the Median, interquartile range [IQR], (min-max); n (%) unless otherwise stated; p-value- Chi-squared test or Fisher’s exact test is cell count<5, median by Mann-Whitney U test. Abbreviations: SMRU Shoklo malaria research unit, ACS antenatal corticosteroids-dexamethasone, EGA estimated gestational age, US ultrasound, PTB preterm birth, n.a not applicable. *Birthweight in live born normal singletons weighed in 72 hours of life. **Neonatal death in live born infants followed up for first 28 days of life.

### Early neonatal and neonatal mortality and associated risk factors

The proportion of early neonatal death was 24.0% (56/233) and neonatal death was 41.1% (69/168) ( Table 2). In multivariable logistic regression models, compared to complete dosing, there was no evidence of benefit of incomplete and no dosing of dexamethasone on early neonatal mortality, as both were associated with elevated early neonatal mortality: adjusted ORs 4.09 (1.39 to 12.00) and 3.13 (1.14 to 8.63), respectively ( Table 3). By contrast, for neonatal death, while there was clear evidence that no dosing was associated with higher mortality, adjusted OR 3.82 (1.42 to 10.27), the benefit of incomplete dosing was uncertain (adjusted OR 1.75 (0.63 to 4.81)) ( Table 3).

**Table 3.** Risk factors associated with early neonatal and neonatal mortality multivariable model.

Covariables		Early Neonatal Death (ENND)				Neonatal Death (NND)				
		Yes	No	Adjusted OR (95%CI)	p	Yes		No	Adjusted OR(95%CI)	P
		n=56	n=177			n=69		n=99		
Congenital abnormality										
	No	45/53 (84.9)	173(97.7)	ref	ref	57/66 (86.4)		98/99 (99.0)	ref	ref
	Yes	8 (15.1)	4 (2.3)	5.34 (1.09-25.99)	0.038	9 /66(13.6)		1/99 (1.0)	14.55 (1.39- 152.07)	0.025
Preterm birth (weeks)										
	28-29	22 (39.3)	17 (9.6)	19.36 (7.19-52.14)	<0.001	25 (36.2)		12 (12.1)	11.27 (4.21- 30.17)	<0.001
	30-31	20 (35.7)	42 (23.7)	4.37 (1.79-10.66)	0.001	25 (36.2)		23 (23.2)	5.03 (2.06- 12.27)	<0.001
	32-33	14 (25.0)	118 (66.7)	ref	ref	19 (27.5)		64 (64.6)	ref	ref
Dexamethasone Doses										
	Complete	10 (17.9)	61 (43.5)	ref	ref	14 (20.3)		32 (32.3)	ref	ref
	Incomplete	18 (32.1)	48 (27.1)	4.09 (1.39-12.00)	0.010	18 (26.1)		33 (33.3)	1.75 (0.63- 4.81)	0.281
	No dose	28 (50.0)	68 (38.4)	3.13 (1.14-8.63)	0.027	37 (53.6)		34 (34.3)	3.82 (1.42- 10.27)	0.008
Smoker										
	No	37 (66.1)	150 (84.7)	ref	ref	49 (71.0)		86 (86.9)	ref	ref
	Yes	19 (33.9)	27 (15.3)	3.36 (1.32-8.53)	0.011	20 (29.0)		13 (13.1)	1.94 (0.73- 5.17)	0.187
Pre-eclampsia										
	No	52 (92.9)	173 (97.7)	ref	ref	65 (94.2)		97 (98.0)	ref	ref
	Yes	4 (7.1)	4 (2.3)	13.77 (2.38-79.62)	0.003	4 (5.8)		2 (2.0)	9.21 (1.9- 71.59)	0.034

Data are presented as n (%) unless otherwise stated.

Congenital abnormality, earlier gestation at birth and pre-eclampsia were significantly and independently associated with an increased risk of neonatal mortality with the addition of smoking in the early neonatal mortality model ( Table 3 and Table 4).

**Table 4.** Risk factors for early neonatal death and neonatal death (NND): Panel A, Panel B. **Panel A. Demographics and morbidity in pregnancy**

Demographics	Early Neonatal death				Neonatal Death			
	Yes	No	Univariable Analysis	p	Yes	No	Univariable Analysis	p
	n=56	n=177	Unadjusted OR (95% CI)		n=69	n=99	Unadjusted OR (95% CI)	
Age, years, Median [IQR]	23.5 [18.25-30.0]	21.0 [19.0-27.5]	1.03 (0.99-1.07)	0.146	23 [18-28]	21 [18-28]	1.01 (0.97-1.06)	0.540
Primigravida, Median [IQR]	21 (37.5)	82 (46.3)	0.70 (0.38-1.29)	0.248	27 (39.1)	54 (54.5)	0.54 (0.29-1.00)	0.050
Refugee (not migrant)	34 (60.7)	93 (52.5)	1.39 (0.76-2.57)	0.286	41 (59.4)	73 (73.7)	0.52 (0.27-1.01)	0.052
Smoker	19 (33.9)	27 (15.3)	2.85 (1.43-5.68)	0.003	20 (29.0)	13 (13.1)	2.70 (1.24-5.89)	0.013
Literate (self-reported) ^	12 (41.4)	63 (54.3)	0.59 (0.26-1.35)	0.215	16/37 (43.2)	31/57 (54.4)	0.64 (0.28-1.47)	0.292
Preterm labour in history, n (%)	6 (10.7)	24 (13.6)	0.77 (0.29-1.98)	0.580	7 (10.1)	12 (12.1)	0.82 (0.31-2.20)	0.819
Neonatal death in history, n (%)	19 (33.9)	60 (33.9)	1.00 (0.53-1.89)	0.997	20 (29.0)	31 (31.3)	0.89 (0.46-1.75)	0.895
1st ANC by 24 weeks, n (%)	42 (75.0)	145 (81.9)	0.66 (0.32-1.35)	0.259	54 (78.3)	83 (83.8)	0.69 (0.32-1.52)	0.361
Anaemic 1st ANC (HCT <30 %), n (%)	11 (19.6)	20 (11.3)	1.92 (0.86-4.30)	0.113	14 (20.3)	12 (12.1)	1.85 (0.80-4.28)	0.154
Morbidity in pregnancy								
Malaria in pregnancy, n (%)	7 (12.5)	26 (14.7)	0.83 (0.34-2.03)	0.682	12 (17.4)	14 (14.1)	1.28 (0.55-2.96)	0.567
Pre-eclampsia, n (%)	4 (7.1)	4 (2.3)	3.33 (0.80-13.77)	0.097	4 (5.8)	2 (2.0)	2.90 (0.53-16.77)	0.214
Antepartum Haemorrhage, n (%)	4 (7.1)	6 (3.4)	2.19 (0.60-8.07)	0.238	5 (7.2)	4 (4.0)	1.86 (0.48-7.18)	0.370
Delivered ≤7 days ACS, n (%)	23/28 (82.1)	95/109 (87.2)	0.68(0.22-2.07)	0.496	26/32 (81.3)	57/65 (87.7)	0.61 (0.19-1.93)	0.399

**Table:** **Panel B. Delivery outcomes**

Birth details		Early Neonatal death				Neonatal death			
		Yes	No			Yes	No		
		n=56	n=177	Unadjusted OR (95%CI)	p	n=69	n=99	Unadjusted OR (95%CI)	p
Place of delivery, n (%)									
	SMRU	41 (73.2)	140 (79.1)	ref	ref	49 (71.0)	82 (82.8)	ref	ref
	home	14 (25.0)	31 (17.5)	1.54 (0.75-3.17)	0.239	18 (26.1)	16 (16.2)	1.88 (0.88-4.03)	0.182
	on the way	1 (1.8)	6 (3.4)	0.57 (0.07-4.86)	0.607	2 (2.9)	1 (1.0)	3.35 (0.30-37.88)	0.329
Male, n (%)	male	38/53 (71.7)	105 (59.3)	1.74 (0.89-3.39)	0.106	47/66 (71.2)	58/99 (58.6)	1.75 (0.90-3.40)	0.100
Congenital Abnormality, n (%)		8/53 (15.1)	4 (2.3)	7.69 (2.22-26.68)	0.001	9 /66(13.6)	1/99 (1.0)	15.47 (1.91-125.31)	0.010
Estimated gestation, Median [IQR]		30.2 [29.1-31.9]	32.4 [31.3-33.3]	0.55 (0.45-0.68)	<0.001	30.4 [29.1-32.1]	32.3 [31.0-33.3]	0.62 (0.50-0.75)	<0.001
Estimated gestation by US, n (%)		47 (83.9)	152 (85.9)	0.86 (0.38-1.97)	0.719	59 (85.5)	85 (85.9)	0.97 (0.40-2.34)	0.972
Preterm birth (weeks), n (%)									
	28-29	22 (39.3)	17 (9.6)	10.91 (4.70-25.30)	<0.001	25 (36.2)	12 (12.1)	7.02 (2.98-16.55)	<0.001
	30-31	20 (35.7)	42 (23.7)	4.01 (1.86-8.66)	<0.001	25 (36.2)	23 (23.2)	3.66 (1.71-7.86)	0.001
	32-33	14 (25.0)	118 (66.7)	ref	ref	19 (27.5)	64 (64.6)	ref	ref
Type of delivery n (%)									
	SVD	52 (92.9)	156(88.1)	ref	ref	65 (94.2)	85 (85.9)	ref	ref
	Breech	3 (5.4)	20 (11.3)	0.45 (0.13-1.58)	0.212	3 (4.3)	13 (13.1)	0.30 (0.08-1.10)	0.07
	Face/forceps	1 (1.8)	1 (0.6)	3.00 (0.18-48.82)	0.44	1 (1.4)	1 (1.0)	1.31 (0.08-21.30)	0.851
Weighed 72 hrs of birth n (%)		47 (100.0)	171 (96.6)	not calculated	0.999	60/60 (100.0)	96/99 (97.0)	not calculated	0.999
Birthweight Median [IQR]		1320 [1160-1600]	1700 [1370-1944]	0.62 (0.50-0.76)	<0.001	1.35 [1.17-1.60]	1.73 [1.42-1.96]	0.997 (0.996-0.998)	<0.001
Dexamethasone Doses, n (%)									
	Complete	10 (17.9)	61 (34.5)	ref	ref	14 (20.3)	32 (32.3)	ref	ref
	Incomplete	18 (32.1)	48 (27.1)	2.29 (0.97-5.41)	0.059	18 (26.1)	33 (33.3)	1.25 (0.53-2.92)	0.612
	No doses	28 (50.0)	68 (38.4)	2.51 (1.13-5.59)	0.024	37 (53.6)	34 (34.3)	2.48 (1.14-5.44)	0.022

^Literacy (self-reported) routinely collected from 2010. Abbreviations: ACS Antenatal corticosteroids, ANC Antenatal clinic, HCT Haematocrit, SMRU Shoklo malaria research unit, US ultrasound, SVD standard vaginal delivery.

### Crude early neonatal and neonatal mortality

The early neonatal and neonatal mortality rate per 1,000 live births for the entire cohort were 240 (95% CI, 190 to 299) and 411 (95% CI, 256 to 417), respectively ( Table 5, Panel A). The largest crude reductions were observed comparing three to zero doses of dexamethasone: 51.7% (95 % CI, 49.9 to 53.5) and 41.7% (95% CI, 40.3 to 42.9), respectively ( Table 5, Panel B).

**Table 5.** Crude early neonatal and neonatal mortality per 1,000 live births (95%CI). **Panel A. Early neonatal and neonatal mortality per 1,000 live births (1 Jan 2008 to 31 Dec 2013).**

Outcomes	Cohort	No dose	Complete(3) doses	Incomplete dose	Any dose
Early Neonatal Death (ENND) 95% CI	240.3 (189.9-299.1)	291.7 (210.2-389.2)	140.8 (78.3-240.2)	272.7 (180.0-390.4)	220.5 (157.2-300.2)
Neonatal Death (NND) 95% CI	410.7 (285.6-417.2)	521.1 (406.8-633.2)	304.3 (190.8-448.0)	352.9 (236.3-490.1)	329.9 (244.4-428.4)

**Table:** **Panel B. Crude % reduction in death (95% CI) by dexamethasone groups (1 Jan 2008-31 Dec 2013).**

Outcomes	Any dose *vs.* No dose	Complete (3) dose *vs.* No dose	Complete (3) dose *vs.* Incomplete dose
Early Neonatal Death (ENND) 95% CI	24.4 (22.8-26.0)	51.7 (49.9-53.5)	48.4 (46.5-50.3)
Neonatal Death (NND) 95% CI	36.7 (35.3-37.9)	41.7 (40.3-42.9)	13.8 (12.6-14.9)

### Neurodevelopmental score

**Figure 3.**
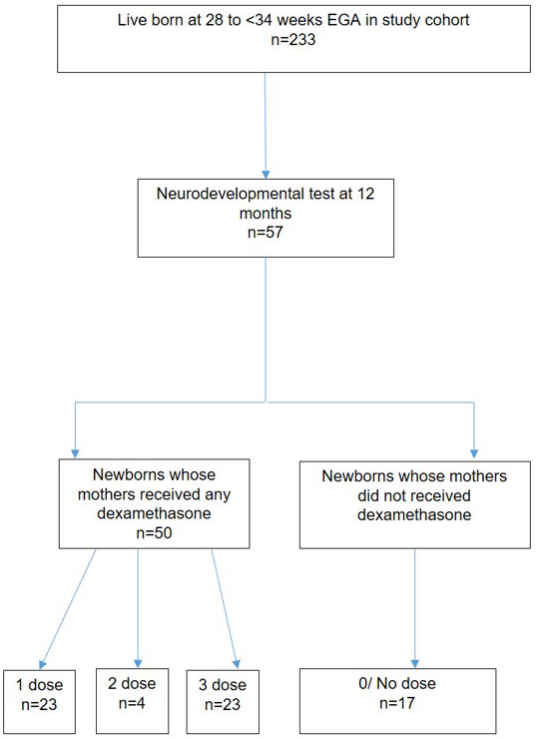
Sub-cohort of infants participating to the Shoklo Developmental test at 12 months of age. EGA, estimated gestational age.

There was no difference observed between the median total score (or separate components including coordination, social, speech and motor milestones) of the Shoklo Developmental test in the sub-cohort ( Figure 3), 28.8% (67/233) of unselected infants at 12 months of age ( Table 6).

**Table 6.** Comparison of Shoklo Neurological Developmental test at different dexamethasone doses: Panel A, and Panel B. **Panel A. Complete *vs.* no dose of dexamethasone.**

Neurological score item	Complete dexamethasone 3 Doses	No Dose	p-value *
	n=23	n=17	
Total Score (n=40)	52.5 [49.0-56.5] (17.0-60.0)	55.0 [49.0-60.0] (42.0-64.0)	0.321
Coordination score	24.0 [22.0-28.0] (8.0-30.0)	26.0 [23.0-30.0] (17.0-35.0)	0.100
Social score	5.0 [5.0-5.5] (1.0-7.0)	5.0 [5.0-6.0] (3.0-7.0)	0.476
Speech score	4.0 [3.0-5.0] (2.0-5.0)	4.0 [4.0-5.0] (2.0-6.0)	0.866
Motor milestones score	19.0 [17.5-20.0] (6.0-23.0)	18.5 [16.0-21.0] (14.0-22.0)	0.931
Total behaviour score ^	14.0 [14.0-15.0] (12.0-15.0)	14.0 [13.0-15.0] (12.0-15.0)	0.813

**Table:** **Panel B. Any number of doses *vs.* no dose of dexamethasone.**

Neurological score item	Any number of dexamethasone Doses,	No Dose	p-value *
	n=50	n=17	
Total Score (n=67)	55.0 [51.0-58.0] (17.0-66.0)	55.0 [49.0-60.0] (42.0-64.0)	0.758
Coordination score	26.0 [24.0-29.0] (8.0-33.0)	26.0 [23.0-30.0] (17.0-35.0)	0.394
Social score	5.0 [5.0-6.0] (1.0-7.0)	5.0 [5.0-6.0] (3.0-7.0)	0.843
Speech score	4.0 [4.0-5.0] (2.0-5.0)	4.0 [4.0-5.0] (2.0-6.0)	0.964
Motor milestones score	19.0 [17.5-20.0] (6.0-23.0)	18.5 [16.0-21.0] (14.0-22.0)	0.718
Total behaviour score ^	14.0 [14.0-15.0] (12.0-15.0)	14.0 [14.0-15.0] (12.0-15.0)	0.665

Data are presented as the median [IQR] and (min-max); *P adjusted for gestation at birth; ^The total behaviour score is not counted towards the total score but provides an indication of the test ‘scene’. A score of 15 (top score) indicates that at the time of the test the infant was interested and not crying or sleep (state of consciousness), in a positive emotional state (happy, smiling) and not afraid of the tester accepting their approach to interact with the tests.

### Maternal febrile morbidity

**Figure 4.**
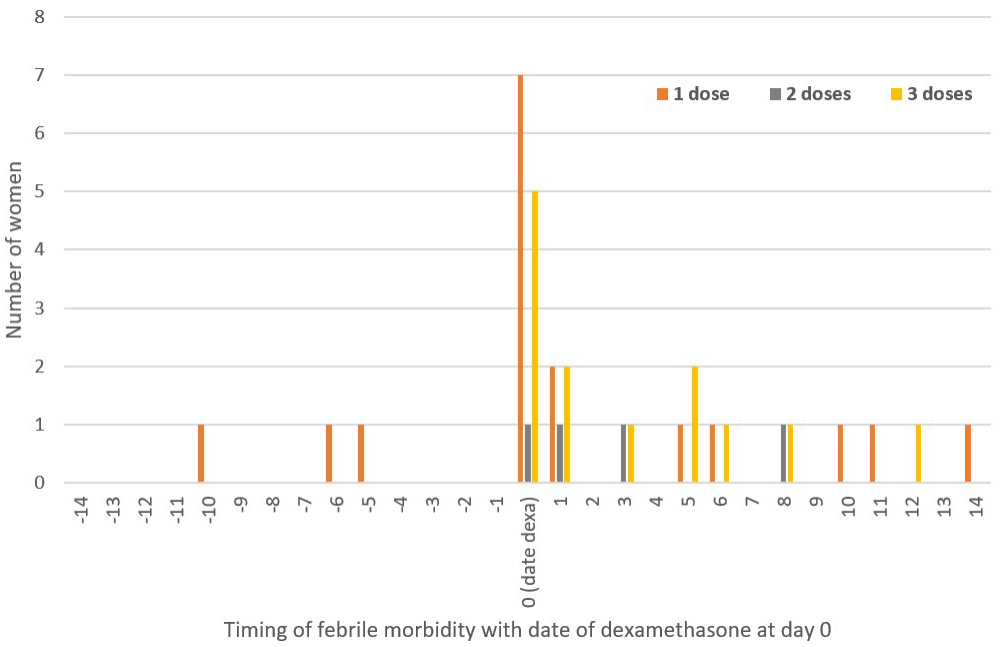
Timing of maternal febrile morbidity according to the date of the first dexamethasone dose.

Among the 144 women who received dexamethasone, 23.6% (34/144) had at least one febrile episode, of which 47.1% (16/34) occurred before receiving dexamethasone, 81.2% (13/16) the day before ( Figure 4). Maternal febrile morbidity after dexamethasone was 8.3% (12/144) by day seven and 12.5% (18/144) by day 14 with a wide range of causes observed.

**Figure 5.**
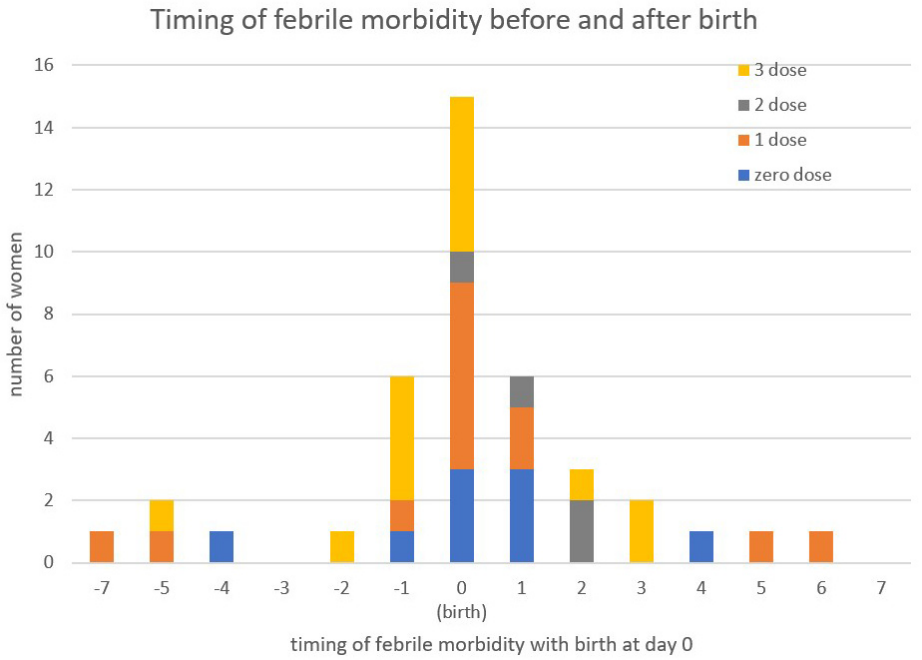
Timing of maternal febrile morbidity seven days either side of the day of birth by dexamethasone group including zero doses.

The proportion of identified fever was lower in those who did not receive dexamethasone, 9.4% (9/96), compared to the dexamethasone treated group, 26.3% (38/144), but the distribution of fever around the birth date was similar ( Figure 5).

## Discussion

In this retrospective study of women who gave birth before 34 weeks, in a low resource setting without assisted ventilatory support, early neonatal (24%) and neonatal mortality (41%) were high. The proportion of neonatal deaths, 30.4% (14 of 46), in the complete dexamethasone group in this setting was higher than observed in the ACTION trial: 19.6% (278 of 1417) in the treatment group and 23.5% (331 of 1406) in the placebo (normal saline) group, where approximately one in five newborns were provided CPAP and a further one in 14 mechanical ventilation ^15^.

In multivariable analysis, zero or incomplete dosing (compared with complete dosing), lower gestation at birth (28-29 or 30-31 weeks compared with 32-33 weeks), congenital abnormality and pre-eclampsia were associated with a significantly increased risk of neonatal death. In this setting, one in five women with a risk of PTB need to receive a complete dexamethasone course to prevent one neonatal death, when compared with one in 25 in the ACTION trial (which included neonates from 26 weeks) ^15^. The role of incomplete dosing remains uncertain. As dexamethasone is one of the only low cost and available tools in conflict affected and fragile states, or amongst marginalized populations where healthcare can be associated with catastrophic expenditure ^34^, this study suggests the benefit in terms of neonatal mortality outweigh potential risks in terms of neurological development at one year, or maternal infection, which is common in tropical South East Asia ^35, 36^.

The ACT Trial suggested upscaling of interventions, including ACS administration in low resource settings, was harmful to preterm neonates in contrast to reports from high income settings ^13^. The ACT trial relied on fundal height and birth weight as a proxy for gestational age, while an obvious strength in this study was that all women had ultrasound: 84% before 24 weeks of gestational age, nearly half in first trimester. As a result, in the ACT trial, ACS exposure occurred in pregnancies where it was not warranted, as the pregnancy was not preterm. Gestation is the single most significant factor to influence neonatal death and reflected in the multivariable analysis presented here. Obstetricians and gynaecologists should take an active leadership role in ensuring implementation of mobile ultrasound to accurately date pregancies, therefore supporting a reduction in health disparities? ^37^. Ultrasound is mobile and technically easier than CPAP to reach more women and can be purposed for roles beyond gestational age assessment, such as identification of multiple pregnancy and placenta praevia, and commencing timely antimalarial prophylaxis.

The ACTION trial ^15^ and a Cochrane systematic review of three cluster-randomized trials, including the ACT trial, suggested a cautious approach to clinical protocols for low-resource settings; one that accounts for both established efficacy and the possibility of adverse effects when certain conditions are not met ^38^. Consequently, WHO has updated its 2015 recommendations on Antenatal corticosteroids, taking into account new evidence from the ACTION trial. While ACS is still recommended between 24 to 34 weeks gestation for women at risk of PTB to improve outcomes, the 2022 update includes CPAP when needed for neonatal respiratory support as a condition ^39^. However, access to assisted ventilation is a frequent unmet need for low-resource settings. Hence this study, unlike the review by Pattanittum *et al*., ^17^ excluded infants born in hospital to maintain a focus on those likely to derive benefit from dexamethasone. Given the challenges in scaling up even bubble CPAP (bCPAP) use in low resource settings ^40^, this study is novel as it demonstrates that a complete course of dexamethasone with no assisted ventilation reduced neonatal mortality in early PTB.

In addition, in the cost-effectiveness analysis of the ACTION Trial, dexamethasone was cost-saving compared with no intervention. Furthermore, sensitivity analysis showed dexamethasone to be cost-saving or highly cost-effective despite the use of ultrasound to confirm gestational age before receiving dexamethasone ^16^. While findings such as these are reassuring, there is a need for further research on the cost-effectiveness of dexamethasone for early PTB in settings such as ours without CPAP or mechanical ventilation.

The main limitation of this study is that not all infants could be followed up to day 28 and only a sub-cohort of infants had neurodevelopmental testing at one year of age. Nevertheless the findings from the Thailand-Myanmar border are consistent with the main outcomes of a Cochrane systematic review of 27 clinical ACS trials (10 in low and middle income settings) published in 2020, and partially fill a gap in knowledge for ACS in understudied and low resource groups ^11^. Another limitation is that the data are from 2008 to 2013, however the current level of neonatal care available to this marginalized population is unchanged and the overall health situation is deteriorating given the current problems with COVID and the *coup d’état* in Myanmar.

## Conclusions

A complete course of dexamethasone for early PTB, in well dated pregnancies screened for maternal infection, in a setting with appropriate newborn care without assisted ventilation, resulted in significant reductions in early neonatal and neonatal mortality. Dexamethasone was not associated with adverse infant neurodevelopmental scores nor an increased risk of morbidity from maternal infection.

## Data Availability

Data cannot be shared publicly as this is a population of undocumented migrants, they have not given their permission to share data. Data are available from the Mahidol-Oxford Research Unit Institutional data access committee (contact Rita Chanviriyavuth, email: datasharing@tropmedres.ac) for researchers who meet the criteria for access to confidential data. Applicants complete an Application Form and a Data Access agreement. Applications are considered by the DAC on a case-by-case basis informed by an assessment criteria, defined in the DAC terms of reference. The type of agreement that applicants are asked to complete will be determined by the DAC. Consideration will involve consultation with PIs, relevant collaborators and other experts. More details are available at https://www.tropmedres.ac/units/moru-bangkok/bioethics-engagement/data-sharing/moru-tropical-network-policy-on-sharing-data-and-other-outputs.
