# Supplementary material for: Antenatal corticosteroids reduce neonatal mortality in settings without assisted ventilatory support: a retrospective cohort study of early preterm births on the Thailand-Myanmar border
Source: Wellcome Open Res. 2024 Mar 14;8:225. Originally published 2023 May 18. [Version 2] doi: 10.12688/wellcomeopenres.19396.2 (PMC11109590; doi:10.12688/wellcomeopenres.19396.2)
Supplement: Peer review history

# Reviewer report: Reviewer response for version 2

**DOI:** 10.21956/wellcomeopenres.23481.r80809

**Published:** 2024-05-09

**Authors:** Kathryn Maitland

**Affiliations:** 1 Kenya Medical Research Institute (KEMRI)-Wellcome Trust Research Programme, Kilifi, Kenya

The authors have satisfactorily addressed the two other reviewers comments. Whilst I agree with both that a limitation of the work presented in the retrospective nature of the design, their findings align with the conclusions of a published Cochrane review on the subject. It is an important step in hypothesis generation in the case for support for a large Phase III trial.

Is the work clearly and accurately presented and does it cite the current literature?

Yes

If applicable, is the statistical analysis and its interpretation appropriate?

Yes

Are all the source data underlying the results available to ensure full reproducibility?

Yes

Is the study design appropriate and is the work technically sound?

Yes

Are the conclusions drawn adequately supported by the results?

Yes

Are sufficient details of methods and analysis provided to allow replication by others?

Yes

Reviewer Expertise:

Paediatrician, global health, clinical trials

I confirm that I have read this submission and believe that I have an appropriate level of expertise to confirm that it is of an acceptable scientific standard.

# Reviewer report: Reviewer response for version 1

**DOI:** 10.21956/wellcomeopenres.21484.r68134

**Published:** 2023-10-23

**Authors:** Lamidi Isah Audu

**Affiliations:** 1 Department of Paediatrics, Kaduna State University, Kaduna, Nigeria; 2 Paediatrics, Federal University Health Sciences Azare, Azare, Bauchi, Nigeria

This manuscript addresses the impact of antenatal corticosteroid on early neonatal and neonatal mortality in a low resource setting and specifically where neonatal respiratory support beyond intranasal oxygen supplementation is not available. It is a retrospective analysis of available records on 240 preterm babies (GA 28-<34 weeks) out of which mothers of 144 received  Dexamethasone (1,2 or 3 doses). The data collection and analysis are clearly and explicitly written. The findings suggest that only full-dose (12mg, 8hrly X 3) is associated with reduction in neonatal mortality.

 The following observations noted would require further comments by the authors:

1. Mothers who received a full dose of Dexamethasone had significantly higher incidence of febrile illness. What was the impact of this on the mothers. This finding is not discussed,
2. Presence of congenital anomaly was a criterion for exclusion but the results show that babies with congenital anomalies were analysed and neonatal mortality was reportedly significantly higher in babies with congenital anomalies. This contradiction should be explained.
3. One of the stated study limitations was the inability to follow up all infants to 28 days, but this information was not stated in the body of the manuscript. it is important to know how many babies were followed up to 28 days as this will unequivocally define the denominator for the determination of the neonatal mortality rate.  

Is the work clearly and accurately presented and does it cite the current literature?

Yes

If applicable, is the statistical analysis and its interpretation appropriate?

Yes

Are all the source data underlying the results available to ensure full reproducibility?

Yes

Is the study design appropriate and is the work technically sound?

Yes

Are the conclusions drawn adequately supported by the results?

Yes

Are sufficient details of methods and analysis provided to allow replication by others?

Yes

Reviewer Expertise:

I am a Neonatologist and a clinical research expert.

I confirm that I have read this submission and believe that I have an appropriate level of expertise to confirm that it is of an acceptable scientific standard.

# Response

**Published:** 2024-03-12

**Authors:** Humayra Aisha Bashir

**Affiliations:** Centre for Tropical Medicine and Global Health, Nu, University of Oxford, Oxford, England, UK

This manuscript addresses the impact of antenatal corticosteroid on early neonatal and neonatal mortality in a low resource setting and specifically where neonatal respiratory support beyond intranasal oxygen supplementation is not available. It is a retrospective analysis of available records on 240 preterm babies (GA 28-<34 weeks) out of which mothers of 144 received  Dexamethasone (1,2 or 3 doses). The data collection and analysis are clearly and explicitly written. The findings suggest that only full-dose (12mg, 8hrly X 3) is associated with reduction in neonatal mortality. We thank the reviewer for their time and helpful suggestions. The following observations noted would require further comments by the authors:

1. Mothers who received a full dose of Dexamethasone had significantly higher incidence of febrile illness. What was the impact of this on the mothers. This finding is not discussed,

The section on maternal fever was reviewed with the data presented in a more logical format given the comments from reviewer 1 and 2. Please see the revised section on Maternal febrile morbidity; no significant difference.

1. Presence of congenital anomaly was a criterion for exclusion but the results show that babies with congenital anomalies were analysed and neonatal mortality was reportedly significantly higher in babies with congenital anomalies. This contradiction should be explained.

Thank you. We have clarified it and now stated instead ‘incidental detection of severe congenital anomaly’ under Exclusion criteria. Under the ultrasound section, we have also made it clearer that experienced sonographers can notice some congenital anomalies.

  

1. One of the stated study limitations was the inability to follow up all infants to 28 days, but this information was not stated in the body of the manuscript. it is important to know how many babies were followed up to 28 days as this will unequivocally define the denominator for the determination of the neonatal mortality rate.  

Amended. In Table 2, the variable Neonatal Mortality is 69/168. We have stated that 168 infants were followed up for the first 28 days of life. We have also amended table 4 to that effect.

# Reviewer report: Reviewer response for version 1

**DOI:** 10.21956/wellcomeopenres.21484.r68132

**Published:** 2023-10-13

**Authors:** Rob Mooji

**Affiliations:** 1 Ndala Hospital, Ndala, Tanzania; 2 OBS/GYN, Erasmus University Rotterdam, Rotterdam, South Holland, The Netherlands

Dear authors,

 Congratulations on this article.

 The exact role of ACT in LMICs is still unclear and this article will help to determine in what settings ACT is beneficial.

 The article is well written with a clear methodology.

 My biggest concern about the article is that is a retrospective cohort study with inherent risk of (selection) bias and you present your results with a degree of certainty which is not supported by this design. So I would advise to formulate your conclusions with more uncertainty and note this in your limitations.

 It is not uncommon in medical research that findings from cohort studies suggest an effect which cannot be confirmed in RCTs. And in this case a selection bias does not seem unlikely: in women with signs of infection doctors might have refrained from ACT. And maybe infected children were born too quick to start or complete ACT.

 On the other hand there seems to be a dosing effect which supports your conclusion that ACT is effective in your setting. And as your setting resembles more the ACTION trial setting than the Althabe trial setting I would expect a positive effect. So your conclusion is probably right, but with this design you can’t state it for certain. You can only conclude that these findings support a positive effect in a setting which has a bit poorer resources than the ACTION trial.

**Introduction**

 The first sentence is a bit confusing, I would add a comma and maybe repeat birth (preterm birth, birth before 37 weeks).

 The 2 ^nd^ sentence is also a bit confusing. 1 million of 15 preterm newborns die.

 The third sentence I can’t find any support for this statement in your references. Of course IRDS is important since it often cannot be treated or prevented (by ACT), but in really poor resource settings I will assume that hypothermia, feeding problems, hypoglycemia and infection are also important, maybe even more (depending on the gestational age). And I feel a lot attention goes to IRDS (and ACT) while more basic aspects of preterm newborn life support are neglected.

 I think IRDS get’s a lot of attention and is assumed to be important, but this is only the case of the level of care is at least enough to treat more simple problems like above.   

 3 ^rd^ paragraph: why is via in italics?

 In this paragraph I would make a bit more clear that the Althabe ACT trial was conducted mostly in health centres. In your discussion you rightly say that early ultrasound makes a big difference, but the difference in obstetric and neonatal care was very big in these 2 settings. In the Althabe trial, besides that the GA was unclear, also infection could often not be ruled out (by laboratory confirmation) and hypoglycemia and hypothermia were not measured, prevented or treated. The ACTION trial was done in LMICs, but the hospitals were much better equipped (I would say not representing 90% of LIC hospitals). So the challenge is to determine what care is essential to allow safety of ACT and it is probably somewhere in between. The WHO was a bit strict in their first prerequisites but this needs to be finetuned. In Tanzania they tried combining ACT with antibiotics, and showed some effect although this was also in a well-equipped teriary centre, so not clear if this would work in health centres as well. https://journals.plos.org/plosone/article?id=10.1371/journal.pone.0193146

**Methods**

 Since the important thing of this article is to determine in which setting ACT is beneficial, you should be very specific about what is and is not possible.

 Page 4: paragraph dexamethasone: did you indicate tocolytics when you suspected infection? What did you use to real this out?

 Results

 I don’t fancy the word “birthed”, but maybe that’s my non-native English.

 2 ^nd^ paragraph: you refer to table 1 that 84,4% of women had ultrasound confirmed GA, bit I think this is Table 2.

 In your discussion you state 84% is US confirmed and add that half is in the 1 ^st^ trimester, but I couldn’t find this in your results section.

**Maternal febrile mortality**

 I am a bit confused by the way you present this. Why bother with the febrile morbidity before the admission of dexamethasone. This should be with the baseline comparison (Table 1) to check if there is any bias (women with fever getting more of less often dexamthason). Febrile morbidity after dexamethasone is relevant, but why not simple state fever after dexamethasone vs after admission (in the non-dexamethasone group)

**Discussion**

 You start by saying you work in a low resource setting. This is of course true, but exactly how low is a bit in the eye of the beholder and a lot of hospitals in LMICs have less resources.

 Like I said before, with a retrospective design be careful to state your results like it is certain, for example when you say NNT of 5.

 I fully agree with the paragraph that probably the most effective modality which can help in making hospitals ready to make safe use of ACT is a ultrasound (more than incubators, ventilators, etc.). You might want to add that in the Althabe trial there was a lot of incorrect use of ACT which diluted the positive effects and exposed more women to the risks. You could also add it is not only availability of the machine, but also affordability of the service, acceptability of the women to attend ANC early.

 The paragraph about ACTION is also nice, and I agree with your idea that probably more children die when thermal care and feeding support are not available than if ventilators are not around. So this a nice thing your study gives information about.

 Can you say something about the 2 outcomes: early and total neonatal death? I can imagine early neonatal death is more related to prematurity and total neonatal death includes more often other causes which explains why only early neonatal mortality is significant. Do you agree, or do you think another reason? It is a finding which is worth mentioning in this section. And why measure the 2 endpoints? Does it say anything about your neonatal care setting that the children who die later are in both groups?

**Limitations**

 Here you really need to say something about the design (retrospective), but you can also say that the partial effect with an incomplete dose suggest your findings are not influenced by bias. And you can say that you think other factors in  neonatal care are more relevant than airway support.

Is the work clearly and accurately presented and does it cite the current literature?

Yes

If applicable, is the statistical analysis and its interpretation appropriate?

Yes

Are all the source data underlying the results available to ensure full reproducibility?

Yes

Is the study design appropriate and is the work technically sound?

Yes

Are the conclusions drawn adequately supported by the results?

Partly

Are sufficient details of methods and analysis provided to allow replication by others?

Yes

Reviewer Expertise:

International maternal health and obstetrics, ACT

I confirm that I have read this submission and believe that I have an appropriate level of expertise to confirm that it is of an acceptable scientific standard, however I have significant reservations, as outlined above.

## References

[1] : A care bundle including antenatal corticosteroids reduces preterm infant mortality in Tanzania a low resource country. *PLoS One* .2018;13(3) : 10.1371/journal.pone.0193146 e0193146 10.1371/journal.pone.0193146 29513706 PMC5841752

# Response

**Published:** 2024-03-10

**Authors:** Humayra Aisha Bashir

**Affiliations:** Centre for Tropical Medicine and Global Health, Nu, University of Oxford, Oxford, England, UK

*Dear authors,*

*Congratulations on this article.*

*The exact role of ACT in LMICs is still unclear and this article will help to determine in what settings ACT is beneficial.*

*The article is well written with a clear methodology.*

*My biggest concern about the article is that is a retrospective cohort study with inherent risk of (selection) bias and you present your results with a degree of certainty which is not supported by this design. So I would advise to formulate your conclusions with more uncertainty and note this in your limitations.*

 We have amended the limitations and conclusion with more uncertainty.

*It is not uncommon in medical research that findings from cohort studies suggest an effect which cannot be confirmed in RCTs. And in this case a selection bias does not seem unlikely: in women with signs of infection doctors might have refrained from ACT. And maybe infected children were born too quick to start or complete ACT.*

*On the other hand there seems to be a dosing effect which supports your conclusion that ACT is effective in your setting. And as your setting resembles more the ACTION trial setting than the Althabe trial setting I would expect a positive effect. So your conclusion is probably right, but with this design you can’t state it for certain. You can only conclude that these findings support a positive effect in a setting which has a bit poorer resources than the ACTION trial.*

 We appreciate these comments to define the setting and context better. They are a helpful perspective and we have tried to address the deficiencies in the first submitted version to better clarify the resources and how the setting compares with ACTION sites in the revised manuscript. See the last paragraph of ‘Settings’ and ‘Neonatal intensive care’ in the methods.

**Introduction**

*The first sentence is a bit confusing, I would add a comma and maybe repeat birth (preterm birth, birth before 37 weeks).*

 Amended as suggested.

*The 2 ^nd^ sentence is also a bit confusing. 1 million of 15 preterm newborns die.*

 Amended as suggested.

*The third sentence I can’t find any support for this statement in your references.*

 Thank you for noting this. We have amended the sentence retaining reference 4 (and removed references 3 and 5).

*Of course IRDS is important since it often cannot be treated or prevented (by ACT), but in really poor resource settings I will assume that hypothermia, feeding problems, hypoglycemia and infection are also important, maybe even more (depending on the gestational age). And I feel a lot attention goes to IRDS (and ACT) while more basic aspects of preterm newborn life support are neglected.*

*I think IRDS get’s a lot of attention and is assumed to be important, but this is only the case of the level of care is at least enough to treat more simple problems like above.*

 Agreed and modified.

*3 ^rd^ paragraph: why is via in italics?*

 No reason, amended.

*In this paragraph I would make a bit more clear that the Althabe ACT trial was conducted mostly in health centres. In your discussion you rightly say that early ultrasound makes a big difference, but the difference in obstetric and neonatal care was very big in these 2 settings. In the Althabe trial, besides that the GA was unclear, also infection could often not be ruled out (by laboratory confirmation) and hypoglycemia and hypothermia were not measured, prevented or treated. The ACTION trial was done in LMICs, but the hospitals were much better equipped (I would say not representing 90% of LIC hospitals). So the challenge is to determine what care is essential to allow safety of ACT and it is probably somewhere in between. The WHO was a bit strict in their first prerequisites but this needs to be finetuned. In Tanzania they tried combining ACT with antibiotics, and showed some effect although this was also in a well-equipped teriary centre, so not clear if this would work in health centres as well.* *https://journals.plos.org/plosone/article?id=10.1371/journal.pone.0193146*

 We have amended the introduction and methods with the help of these comments (and added the reference into the introduction).

**Methods**

*Since the important thing of this article is to determine in which setting ACT is beneficial, you should be very specific about what is and is not possible.*

 We have clarified the setting further in the methods.

*Page 4: paragraph dexamethasone: did you indicate tocolytics when you suspected infection? What did you use to real this out?*

 We have amended the section on page 4 in the methods about tocolytics and investigation of maternal fever.

**Results**

*I don’t fancy the word “birthed”, but maybe that’s my non-native English.*

 And we have frequently received the opposite comment for delivered. One reviewer (not this manuscript) suggested that ‘delivered’ sounds more like getting a letter or a parcel. If the reviewer does not mind we prefer to continue with “birthed”.

*2 ^nd^ paragraph: you refer to table 1 that 84,4% of women had ultrasound confirmed GA, bit I think this is Table 2.*

 Amended and thank you for pointing it out.

*In your discussion you state 84% is US confirmed and add that half is in the 1 ^st^ trimester, but I couldn’t find this in your results section.*

 It is in the section here: “The median maternal age was 22 years, almost half (43.8%, 105/240) were primigravidae, 47.5% (114/240) attended first ANC visit in first trimester and most women had ultrasound confirmed gestation (84.4%, 204/244) ( Table 2).

**Maternal febrile mortality**

*I am a bit confused by the way you present this. Why bother with the febrile morbidity before the admission of dexamethasone. This should be with the baseline comparison (Table 1) to check if there is any bias (women with fever getting more of less often dexamethasone). Febrile morbidity after dexamethasone is relevant, but why not simple state fever after dexamethasone vs after admission (in the non-dexamethasone group)*

 We agree that the clarity of this section needs to improve. We have taken your suggestions to examine fever in the first 72 hours from birth. We have deleted Figure 5.

**Discussion**

*You start by saying you work in a low resource setting. This is of course true, but exactly how low is a bit in the eye of the beholder and a lot of hospitals in LMICs have less resources.*

 Agree and we have amended the last paragraph of the “settings” in the methods and rephrased the discussion.

*Like I said before, with a retrospective design be careful to state your results like it is certain, for example when you say NNT of 5.*

 Agree and we have amended the statement.

*I fully agree with the paragraph that probably the most effective modality which can help in making hospitals ready to make safe use of ACT is a ultrasound (more than incubators, ventilators, etc.). You might want to add that in the Althabe trial there was a lot of incorrect use of ACT which diluted the positive effects and exposed more women to the risks. You could also add it is not only availability of the machine, but also affordability of the service, acceptability of the women to attend ANC early.*

 Agree and amended.

*The paragraph about ACTION is also nice, and I agree with your idea that probably more children die when thermal care and feeding support are not available than if ventilators are not around. So this a nice thing your study gives information about.*

 Thank you.

*Can you say something about the 2 outcomes: early and total neonatal death? I can imagine early neonatal death is more related to prematurity and total neonatal death includes more often other causes which explains why only early neonatal mortality is significant. Do you agree, or do you think another reason? It is a finding which is worth mentioning in this section. And why measure the 2 endpoints? Does it say anything about your neonatal care setting that the children who die later are in both groups?*

 Again we thank you for the care you have taken as a reviewer. In the first submission, we used all infants followed to day 7 for early neonatal death (n=233) and all followed to day 28 for neonatal death (n=168) – so we were not comparing the same population. Table 5 has been replaced so the denominator is the same i.e. infants followed to day 28. We were interested to understand if ACS helped survival in terms of acute RDS (Early NND) but infants just died later (NND) but this does not appear to be the case.

**Limitations**

*Here you really need to say something about the design (retrospective), but you can also say that the partial effect with an incomplete dose suggest your findings are not influenced by bias. And you can say that you think other factors in  neonatal care are more relevant than airway support.*

 Agree and amended.

 Is the work clearly and accurately presented and does it cite the current literature? Yes Is the study design appropriate and is the work technically sound? Yes Are sufficient details of methods and analysis provided to allow replication by others? Yes If applicable, is the statistical analysis and its interpretation appropriate? Yes Are all the source data underlying the results available to ensure full reproducibility? Yes Are the conclusions drawn adequately supported by the results? Partly

 References 1. Massawe A, Kidanto HL, Moshiro R, et al.: A care bundle including antenatal corticosteroids reduces preterm infant mortality in Tanzania a low resource country. PLoS One. 2018; 13 (3): e0193146 PubMed Abstract | Publisher Full Text
